# Thin Ga_2_O_3_ Layers by Thermal Oxidation of van der Waals GaSe Nanostructures for Ultraviolet Photon Sensing

**DOI:** 10.1021/acsanm.4c02685

**Published:** 2024-07-31

**Authors:** Nathan D. Cottam, Benjamin T. Dewes, Mustaqeem Shiffa, Tin S. Cheng, Sergei V. Novikov, Christopher J. Mellor, Oleg Makarovsky, David Gonzalez, Teresa Ben, Amalia Patanè

**Affiliations:** † School of Physics and Astronomy, University of Nottingham, Nottingham NG7 2RD, United Kingdom; ‡ University Research Institute on Electron Microscopy and Materials, IMEYMAT, Universidad de Cadiz, 11510 Cadiz, Spain

**Keywords:** gallium selenide, van der Waals crystals, gallium oxide, oxidation, UV-photonics

## Abstract

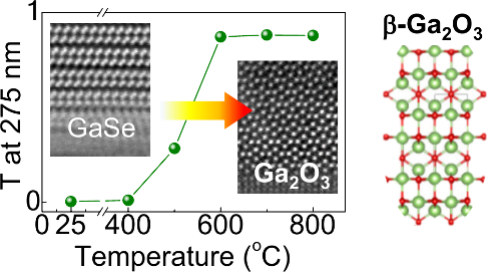

Two-dimensional semiconductors (2DSEM) based on van der Waals crystals offer important avenues for nanotechnologies beyond the constraints of Moore’s law and traditional semiconductors, such as silicon (Si). However, their application necessitates precise engineering of material properties and scalable manufacturing processes. The ability to oxidize Si to form silicon dioxide (SiO_2_) was crucial for the adoption of Si in modern technologies. Here, we report on the thermal oxidation of the 2DSEM gallium selenide (GaSe). The nanometer-thick layers are grown by molecular beam epitaxy on transparent sapphire (Al_2_O_3_) and feature a centro-symmetric polymorph of GaSe. Thermal annealing of the layers in an oxygen-rich environment promotes the chemical transformation and full conversion of GaSe into a thin layer of crystalline Ga_2_O_3_, paralleled by the formation of coherent Ga_2_O_3_/Al_2_O_3_ interfaces. Versatile functionalities are demonstrated in photon sensors based on GaSe and Ga_2_O_3_, ranging from electrical insulation to unfiltered deep ultraviolet optoelectronics, unlocking the technological potential of GaSe nanostructures and their amorphous and crystalline oxides.

## Introduction

The past couple of decades have seen the discovery and development of two-dimensional semiconductors (2DSEM) based on van der Waals (vdW) crystals.^1−3^ These materials have generated an enormous amount of interest for their unique and tunable electronic properties with potential for an extensive range of applications in nanoelectronics.^4,5^ The controlled oxidation of these emerging semiconductors holds great importance for different technologies, from surface passivation to dielectric insulation in electronic and optoelectronic components. Thermal oxidation of Si has represented one of the most important advances in modern electronics. Through high-temperature annealing, oxygen molecules react with Si atoms, resulting in the formation of an amorphous SiO_2_ layer.^6^ This oxide layer acts as a dielectric gate or protective mask during semiconductor manufacturing processes. Implementing a similar approach with 2DSEM is pivotal for exploiting and advancing their emerging applications, including 2D device architectures via patterned oxide and semiconducting layers.

Here, we report on the formation of the crystalline oxide Ga_2_O_3_ via post-growth conversion of thin layers of the vdW semiconductor GaSe by thermal annealing in a controlled, oxygen-rich environment. Oxygen chemisorption and physisorption can be energetically favorable in GaSe, particularly in the presence of crystal defects, such as Se-vacancies.^7^ In particular, the Se-atoms lone-pair states are located close to the Fermi level at the top of the valence band, making the surface sensitive to external adsorbates, such as oxygen and water. The thermal oxidation of bulk GaSe has been examined in the literature, revealing an intriguing evolution of the GaSe surface for increasing temperatures of up to ≈700 °C.^8^ However, present studies focus on bulk crystals that can only partially oxidize^8^ or on exfoliated flakes.^9^ The controlled thermal oxidation of a scalable thin film to create a crystalline thin oxide layer has not yet been demonstrated or exploited. This is important to examine the thermal stability of GaSe nanostructures in oxygen; to explore a route for the realization of a GaSe/Ga_2_O_3_ junction; and to realize high-quality thin layers of Ga_2_O_3_ that are challenging to produce by other techniques.

**Figure 1:**
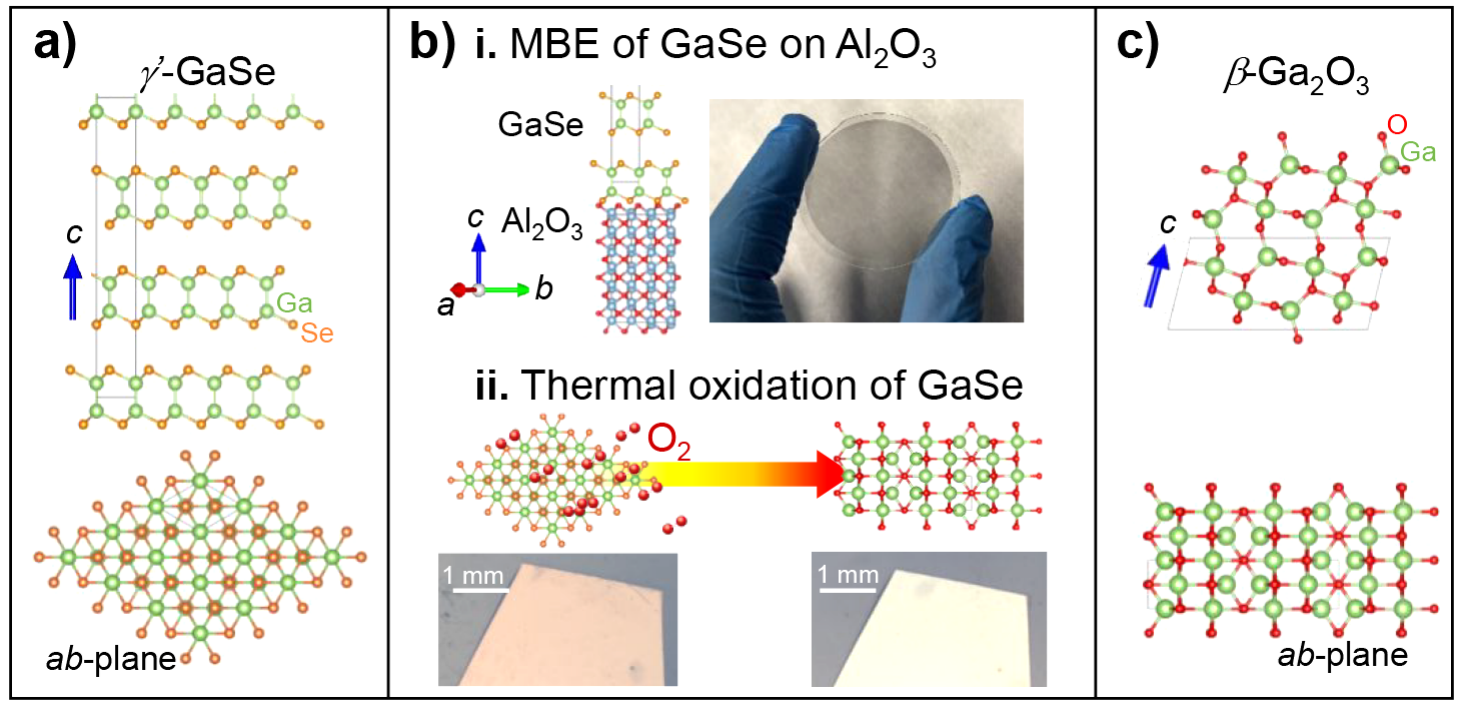
Chemical conversion of MBE-grown GaSe into Ga_2_O_3_. (a). Cross-sectional view (top) and planar view (bottom) of the crystal structure of γ′-GaSe. (b) Photograph and schematic of (i) MBE γ′-GaSe on Al_2_O_3_ and (ii) optical microscopy images of the surface of γ′-GaSe and β-Ga_2_O_3_. The inset in panel (ii) is a schematic of the changes during thermal oxidation of γ′-GaSe and its conversion into β-Ga_2_O_3_. (c) Schematic of the cross-sectional view (top) and planar view (bottom) of the crystal structure of β-Ga_2_O_3_.

Top-down approaches to the fabrication of thin GaSe layers (*e.g*. exfoliation of bulk crystals by “scotch-tape”) for thermal oxidation present drawbacks: the exfoliation of bulk crystals produces only small area (<100 μm^2^) flakes; also, the properties and stability of the layers can be affected by their exposure to chemical species (e.g., solvent and polymers) during exfoliation. On the other hand, epitaxial growth is a promising technique to realize large-scale, high-quality crystals, overcoming the reliance on exfoliated flakes.^10^ To date, various techniques have been used for GaSe epitaxy, such as molecular beam epitaxy (MBE)^11−13^ and chemical vapor deposition (CVD).^14^ These recent advances in epitaxial growth of GaSe thin-films make high quality crystals readily accessible for studies of post-growth processing and oxidation at scale. This work demonstrates the chemical conversion of thin layers of GaSe grown by MBE into crystalline Ga_2_O_3_ (Figure 1a-b-c), as probed by comprehensive studies of the chemical composition, crystallinity, and optical properties of the layers. The scalable conversion of GaSe into an oxide offers a promising pathway for tailored optical properties, including optical constants and transmission in both visible and ultraviolet (UV) spectral ranges. Previously, thin-film oxides, such as crystalline and amorphous Ga_2_O_3_, have garnered a tremendous amount of interest as potential candidates to surpass the power device performance of current SiC and GaN technologies,^15−17^ and UV photonics.^18−20^ In particular, among materials for UV sensing, Ga_2_O_3_ has emerged as an ideal candidate in the UV–C band (200–280 nm) due to its large bandgap (4.5–5.3 eV).^18,21^ The UV–C band features a number of advantages for low noise optical communication as it is free of solar background; also, it is compatible with different detection geometries (i.e., nonline-of-sight and line-of-sight).^22,23^ Thus, our findings on the fabrication of high-quality nanostructures by epitaxy and post-growth oxidation offer opportunities for a plethora of important applications.

## Results and Discussion

### Chemical Conversion of GaSe into Ga_2_O_3_

**Figure 2:**
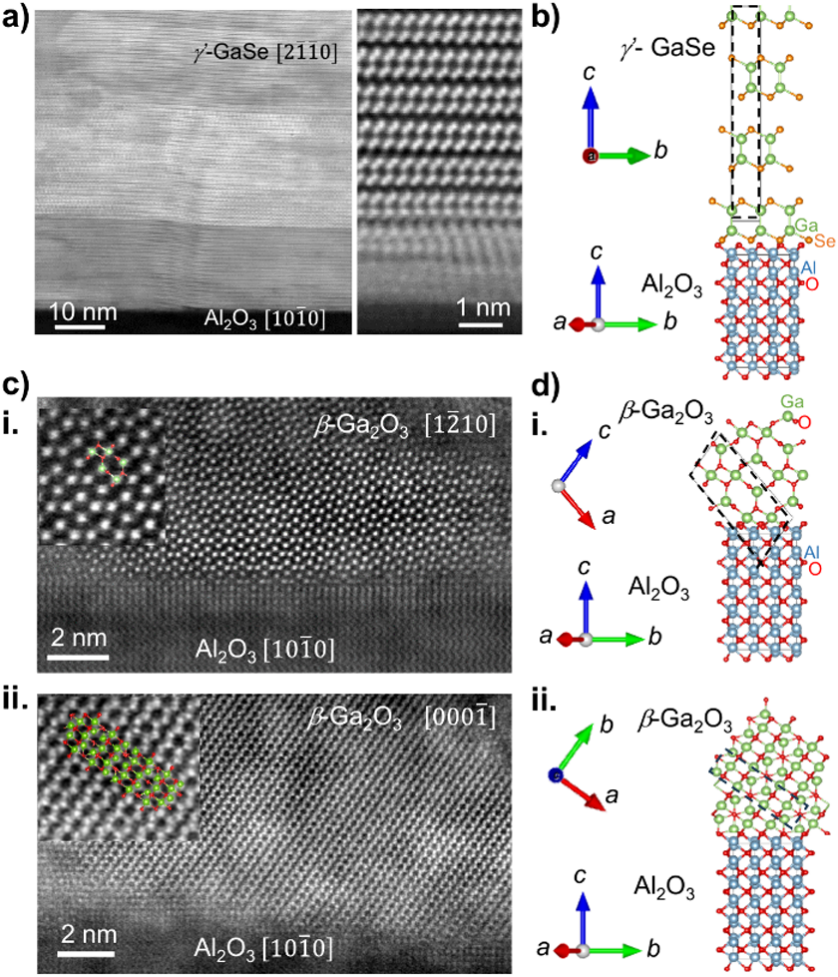
Crystal structures and interfaces of γ′-GaSe and β-Ga_2_O_3_ with sapphire. (a) Cross-sectional high resolution (HR) high-angle annular dark-field scanning transmission electron microscopy (HAADF-STEM) images of as-grown γ′-GaSe on sapphire (thickness *l* = 75 nm). (b) Schematic of the interface of γ′-GaSe on Al_2_O_3_. (ci-ii) HR-STEM images showing the formation of coherent interfaces for 2 different crystallographic orientation relationships (COR) of a sample annealed at *T*_*a*_ = 900 °C for *t*_*a*_ = 30 min in an Ar/O_2_ mixture. The superposition of the crystal structures with different orientations of β-Ga_2_O_3_ on the enlarged ADF images in the insets show a complete fitting. (di-ii) Schematic of the interface of β-Ga_2_O_3_ on Al_2_O_3_, as observed in the ADF images for both CORs.

Ga_2_O_3_ can exist in five distinct polymorphs (α, β, γ, δ, and ε), of which β-Ga_2_O_3_ is the most thermodynamically stable.^24−26^ While well studied in the bulk form, Ga_2_O_3_ is yet to be fully explored as a thin-film.^27−33^ This is in part due to the difficulty in growing high-quality thin layers using traditional melt-growth techniques, such as floating zone, Czochralski,^27,28^ edge-defined film-fed growth^29^ and the Bridgman method.^30^ Emerging methods employed to produce thin-film metal oxides include the mechanical exfoliation of the native oxide formed at a metal-gas interface (e.g., in Al_2_O_3_)^34^ or liquid metal deposition and subsequent native oxidation, as demonstrated for Ga_2_O_3_.^35^ However, these methods rely on formation of the native oxide and thus are limited to 2D films of a few nanometer thickness. Here, high-quality wafer-scale GaSe crystals with a controllable range of layer thicknesses, *l*, from 24 to 75 nm were grown by MBE on 2-inch *c*-plane (0001) sapphire (Al_2_O_3_) wafers. The grown GaSe layers feature a dominant D_3d_ polymorph, referred to as γ′-GaSe (Figure 1a-b).^12^ Thermal annealing was performed in a tube furnace under a controlled atmosphere of oxygen (0.5 sL/min) and argon (2.0 sL/min) (details in Methods). Using a range of annealing temperatures *T*_*a*_ (from 400 to 900 °C) and annealing times *t*_*a*_ (from minutes to hours), we achieved the sequential conversion of GaSe into an intermediate Ga_2_Se_3_ phase, followed by conversion into amorphous Ga_2_O_3_, and ultimately the formation of crystalline β-Ga_2_O_3_ (Figure 1b-c and Figure 2).

The crystal structure of our MBE-grown thin layers of GaSe on Al_2_O_3_ (before any post-growth thermal annealing) was studied by high resolution high angle annular dark field scanning transmission electron microscopy (HR-HAADF-STEM). Figure 2a and Figure S1 in the Supporting Information (SI) show cross-sectional images of the interface along the [1010] axis of the Al_2_O_3_ substrate. The GaSe epitaxial layers consist of nanometer-scale grains that are aligned along the growth direction [0001] but misaligned in the growth plane. Each vdW layer comprises a centrosymmetric tetralayer (cTL) with the Se atoms of the upper layer of the cTL rotated by 60° relative to the Se atoms of the lower layer. The polytype identified is consistent with the γ′-phase (space group D_3d_), which is characterized by the stacking of three cTLs translated by one-third along the <1010> type directions. The predominant crystallographic orientation relationship (COR) for the Al_2_O_3_/GaSe interface in the images is Al_2_O_3_[1010]∥GaSe[2110] (zone axis) and Al_2_O_3_[0001]∥GaSe[0001]. Figure 2b illustrates the atomic structure of the interface using this COR. The lack of an optimal match between the lower Se-monolayer of GaSe and the upper O-monolayer of the Al_2_O_3_ surface accounts for the absence of coherent interfaces observed in the atomic column resolution images.

The TEM images and energy dispersive X-ray diffraction (EDX) elemental maps show that for *T*_*a*_ > 600 °C, thin-layers of GaSe are transformed by the oxidation reaction with the Se-atoms replaced by oxygen. The EDX analysis of the as-grown GaSe layers reveals a well-defined stoichiometric [Ga]/[Se] ratio of 1 (Figure S2 in SI). Following a thermal oxidation of GaSe at *T*_*a*_ = 600 °C, the [Ga]/[O] ratio changes to 2/3 and the concentration of elemental Se is negligible within the experimental error of the technique (Figure S3 in SI). At these low anneal temperatures (*T*_*a*_ = 600 °C), the GaSe layer is transformed into a predominant amorphous oxide (a-Ga_2_O_3_). However, following a thermal oxidation at higher temperatures (e.g., *T*_*a*_ = 700, 800, and 900 °C), the GaSe film is transformed into a crystalline oxide, corresponding to β-Ga_2_O_3_, which is the most thermodynamically stable phase of Ga_2_O_3_. Figure 2c depicts representative micrographs of the structure, in which β-Ga_2_O_3_ (space group *C*2/*m*) is identified from their fast Fourier transform (FFT) patterns. The insets in Figure 2ci and 2cii show the perfect overlap of the structural model with the atomic columns in the zoomed HAADF-STEM image. As can be seen in the image of Figure 2ci, the interface between the oxide grain and the Al_2_O_3_ substrate is coherent and no mismatch defects are present. The COR in this image between the sapphire and the gallium oxide are Al_2_O_3_[1010]∥Ga_2_O_3_[1210] (zone axis) and Al_2_O_3_(0001)∥Ga_2_O_3_ (2021). The atomic model of the interface using this COR (Figure 2di) shows the perfect alignment between the phases. As shown in Figure 2cii and dii, other CORs can also be observed corresponding to coherent Al_2_O_3_/Ga_2_O_3_ interfaces.

In principle, a perfect GaSe crystal should be inert to O_2_ at room temperature as the processes of dissociation and adsorption of O_2_ on the GaSe surface are not thermodynamically favorable.^36^ However, the chemical reactivity of GaSe changes in the presence of O_2_^–^ anions generated either by light-induced electron transfer^36^ or in the presence of defects, such as grain boundaries and Se-vacancies.^7^ These vacancies tend to be filled with O atoms; also, due to the smaller nuclei radius and greater electronegativity of O compared to Se, the Ga–Se bonds around the vacancy are weakened, thus triggering further oxidation. These processes are accelerated at high temperatures due to the increased reaction rate and desorption of Se from the GaSe surface. Our data indicate that the oxidation of the uppermost layers does not prevent the penetration of O atoms deeper into the crystal. Thus, contrary to previous reports showing the partial oxidation of GaSe by the formation of Ga_2_Se_3_/Ga_2_O_3_ in bulk GaSe crystals at high temperatures,^8^ here we can achieve the full conversion of thin layers of GaSe into an oxide. As the interface of the oxide layer progresses into the GaSe layer, the volume of the crystal should contract. Using the density and molecular weights of GaSe and Ga_2_O_3_, we estimate the contraction of the layer thickness to be about 46% (*l*_Ga2O3_*≈* 0.54*l*, see details in SI2). However, the overall contraction of the layer thickness derived from the analysis of the TEM images is smaller (∼15%). The explanation for this discrepancy can be found in the presence of voids in the oxide layer. The voids are buried below the surface and form a porous, thin film embedded within the Ga_2_O_3_ layer (Figure S1 in the SI), making up over 20% of the total volume. Similar voids can form in other semiconductors, such as amorphous-Si.^37^ In the sections below we examine the changes in the optical and vibrational properties of the layers as they are annealed under increasing temperatures.

### Optical Properties of GaSe and Ga_2_O_3_

**Figure 3:**
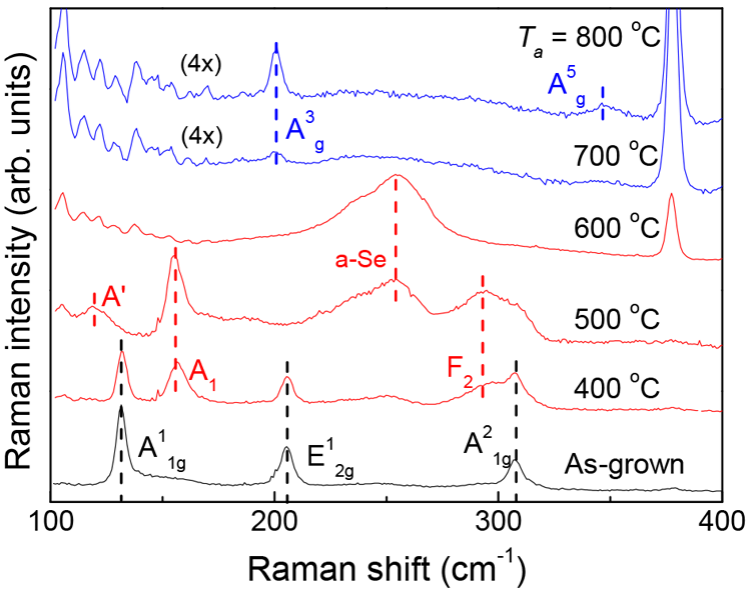
Conversion of GaSe into Ga_2_O_3_ probed by Raman spectroscopy. Raman spectra of as-grown γ′-GaSe (thickness *l* = 75 nm) and samples thermally annealed in an oxygen atmosphere at different temperatures *T*_*a*_ and annealing time *t*_*a*_ = 30 min. The Raman modes for GaSe (black), Ga_2_Se_3_ (red), and Ga_2_O_3_ (blue) are labeled by dashed lines. Curves in blue and red correspond to spectra with peaks from β-Ga_2_O_3_, and Ga_2_Se_3_/amorphous-Se (peak at 250 cm^–1^), respectively. The peak at 377 cm^–1^ arises from the sapphire substrate and is dominant following the formation of β-Ga_2_O_3_, which is optically transparent under the laser excitation used in the experiment (λ = 532 nm).

The Raman shift of the characteristic vibrational modes of a thin layer are finger prints of specific compositions and crystal structures. Thus, Raman spectroscopy and imaging provide an effective, non-destructive means of probing as-grown and oxidized nanometer-thick layers, and their uniformity over wafer-scale samples. Figure 3 shows the Raman spectra of an MBE-grown GaSe layer (*l* = 75 nm) before and after annealing at temperatures ranging from *T*_*a*_ = 400 to 800 °C and annealing time *t*_*a*_ = 30 min. In the pristine layers, the A^1^_1g_, E^1^_2g_ and A^2^_1g_ peaks of GaSe are observed at 132 cm^–1^, 206 and 308 cm^–1^, respectively, as reported for the centrosymmetric (D_3d_) polymorph of GaSe.^12^ The annealing at *T*_*a*_ = 400 °C produces additional peaks at 156 and 293 cm^–1^, suggesting partial conversion of the GaSe layers into Ga_2_Se_3_.^38^ Increasing the annealing temperature to *T*_*a*_ = 500 °C promotes a full conversion of GaSe into Ga_2_Se_3_, as indicated by the vanishing intensity of the GaSe modes. At *T*_*a*_ = 600 °C, only the Raman modes for crystalline (237 cm^–1^) and amorphous-Se (250 cm^–1^) are observed, suggesting that the GaSe layer fully converts into an amorphous oxide.^9,39^ Annealing at *T*_*a*_ = 700 °C produces a Raman peak at 201 cm^–1^ corresponding to the A^3^_g_ mode of *β-*Ga_2_O_3_ as the oxide begins to crystallize; finally, increasing further the temperature to *T*_*a*_ = 800 °C enhances this mode and reveals the A^5^_g_ mode of *β-*Ga_2_O_3_ (347 cm^–1^).

**Figure 4:**
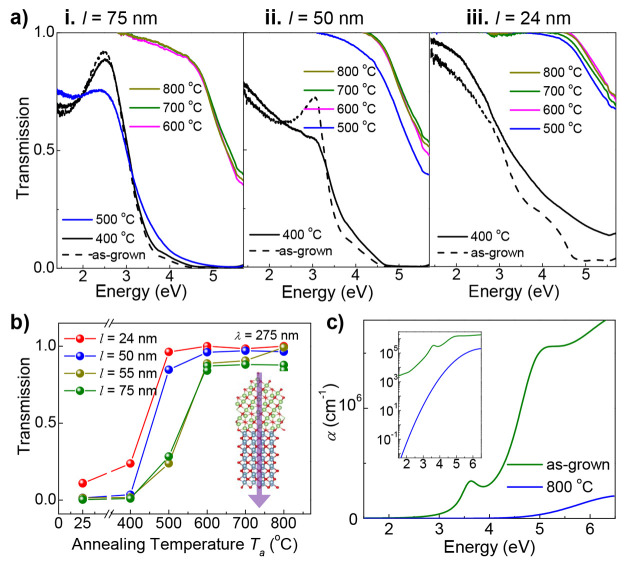
Tunable optical transmission by conversion of GaSe into Ga_2_O_3_. (a) Transmission spectra at room temperature of as-grown γ′-GaSe of thickness (i) *l* = 75 nm, (ii) 50 nm, and (iii) 24 nm and of the same materials after thermal annealing in oxygen at different temperatures *T*_*a*_ and time *t*_*a*_ = 30 min. (b) Transmission at λ = 275 nm versus *T*_*a*_ for γ′-GaSe samples of different thickness *l*. Inset: Optical transparency by conversion of GaSe into Ga_2_O_3_ at high *T*_*a*_ and λ = 275 nm. (c) Absorption (α) spectra, as determined by ellipsometry for as-grown GaSe (*l* = 75 nm) and the same material annealed in oxygen at *T*_*a*_ = 800 °C. Inset: Absorption spectra with a log-scale for the *y*-axis.

The Raman data illustrate that as the annealing temperature increases the oxidation of GaSe progresses via different reactions:^40,41^
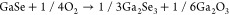
1
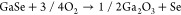
2
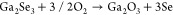
3leading to the formation of an oxide at *T*_*a*_ ≥ 600 °C and other byproducts, such as Ga_2_Se_3_ and amorphous-Se, at lower *T*_*a*_. The changes in the Raman spectra are paralleled by an increased surface roughness with increasing *T*_*a*_, as probed by atomic force microscopy (AFM) (Figure S4 in the SI), and by changes in the optical transmission, as shown in Figure 4a for different film thicknesses (*l* = 24, 50, and 75 nm).

The optical transmission of the 75 nm-thick as-grown GaSe (dashed line in Figure 4ai) shows a steady decrease over the spectral range *hv* = 2.5–3.5 eV, with effectively no transmission above *hv* = 4.5 eV. The transmission measured for *T*_*a*_ = 400 °C is similar to that of the as-grown sample, indicating little change of the material, in line with the Raman study. Increasing *T*_*a*_ to 500 °C causes a more significant change, likely, as the Raman suggests, due to the formation of Ga_2_Se_3_ and amorphous-Se. Finally, for *T*_*a*_ ≥ 600 °C the threshold energy for optical transparency shifts to about *hv* = 4.5 eV. For the intermediate low- *T*_*a*_ stages, it is not possible to distinguish clearly the transmission due to the amorphous a-Ga_2_O_3_ and crystalline β-Ga_2_O_3_.

Samples with different layer thickness *l* show a similar dependence of the transmission spectra on the annealing temperature (Figure 4ai-ii-iii). However, a stronger modification of the transmission spectrum is observed in thinner layers as *T*_*a*_ approaches 500 °C. In contrast, the dispersion of the transmission curves for samples annealed at *T*_*a*_ ≥ 600 °C is relatively independent of *l* (excluding the overall reduction in transmission in thicker layers). This is shown in Figure 4b, which plots the optical transmission at λ = 275 nm (*hv* = 4.5 eV) versus *T*_*a*_ (*t*_*a*_ = 30 min) for all samples. Here, the transition from GaSe to Ga_2_O_3_ manifests with a clear change in the transmission at a characteristic temperature between *T*_*a*_ = 450 °C and *T*_*a*_ = 600 °C that depends on *l*: the thicker layers require higher temperatures to become fully transparent in the visible spectral range.

Studies of the thermal oxidation of GaSe at different annealing times *t*_*a*_ (from minutes to hours) did not reveal any significant difference in the optical transmission. This indicates that the Deal-Grove model^6^ describing the linear increase in the oxide thickness with increasing oxidation time does not apply to thin layers. This behavior, also reported for Si, is attributed to a non-Fickian oxygen diffusion.^42^ As for Si, the thermal oxidation of a thin layer is a dynamic process in which the oxidant migration and reaction kinetics are strongly influenced by the stress–strain differences within the oxide. For GaSe, the oxidation progresses via the formation of different byproducts (e.g., Ga_2_Se_3_, amorphous-Se, crystalline and amorphous Ga_2_O_3_) and a significant contraction of the lattice. Also, the oxidation behavior of thin layers grown by MBE can differ from that of single bulk crystals^8^ due to the different crystal structures.

The absorption (α) spectra and the optical constants of as-grown GaSe (*l* = 75 nm) and Ga_2_O_3_, as derived by ellipsometry (details in SI4), are compared in Figure 4c and Figure S5 in the SI. The spectra in Figure 4c show a lower absorption coefficient in the oxide across a wide spectral range and a clear shift of the absorption edge from *hv* = 2.3 eV in GaSe to *hv* = 4.5 eV in Ga_2_O_3_. For GaSe, the low transmission at high photon energies arises from the large absorption coefficient: for α = 7.8 × 10^5^ cm^–1^ at *hv* = 4.5 eV, the absorption length is 1/α = 12.8 nm, smaller than the GaSe layer thickness. For Ga_2_O_3_, α = 10^3^ cm^–1^ at *hv* = 4.5 eV corresponding to a much larger absorption length (1/α = 100 μm). Due to their wide bandgap, both amorphous and crystalline Ga_2_O_3_ are transparent across a broad spectrum that extends from UV to visible wavelengths.

### Electrical Conductivity and UV–C Sensing in Amorphous and Crystalline Ga_2_O_3_

**Figure 5:**
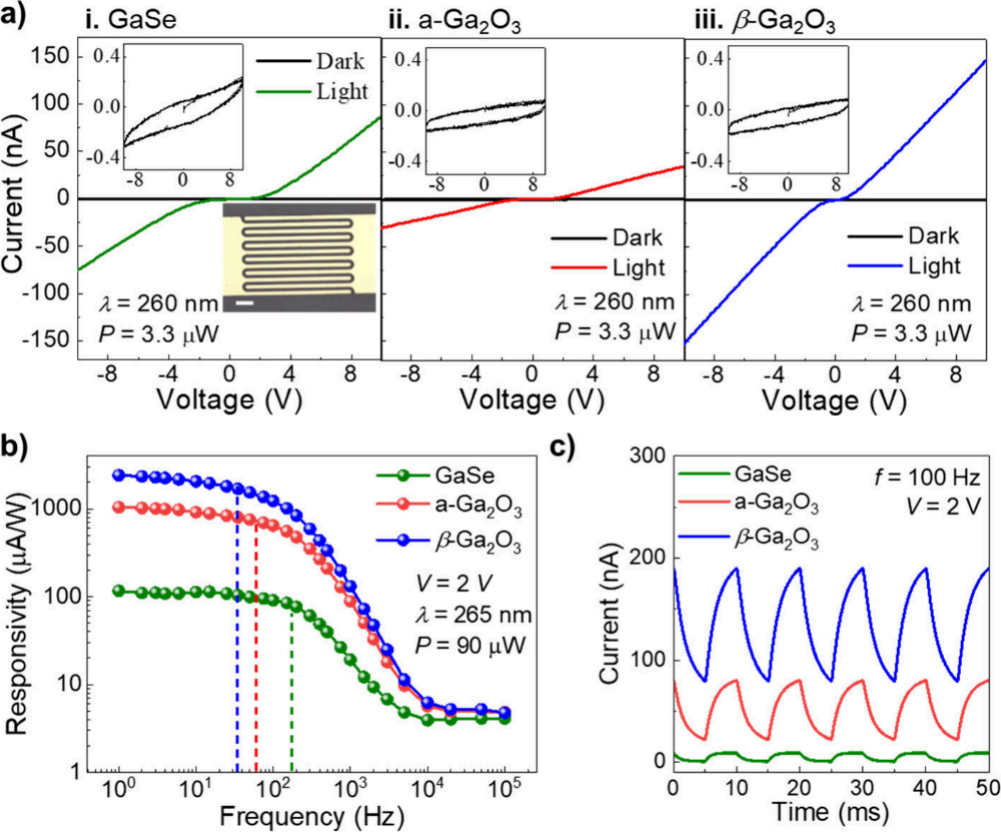
UV–C sensing. (a) Current–voltage curves at room temperature in the dark (black line) and under excitation with photons of wavelength λ = 260 nm (*P* = 3.3 μW, colored lines) of a photon sensor based on (i) 75 nm-thick GaSe, (ii) a-Ga_2_O_3_ (*T*_*a*_ = 600 °C), and (iii) β-Ga_2_O_3_ (*T*_*a*_ = 800 °C). The insets are zoomed-in figures of the current–voltage curves in the dark. Inset in panel (i): Optical image of a typical device. Scale bar (bottom left): 1 mm. (b) A comparison of the frequency response of the photoresponsivity of the devices in panel (a) under UV–C LED excitation (λ = 265 nm, *P* = 90 μW, *V* = 2 V). Dashed lines show the 3 dB frequency bandwidth. (c) Temporal modulation of the current at a set frequency (*f* = 0.1 kHz) for the devices shown in panel (a) (*V* = 2 V, λ = 265 nm, *P* = 90 μW).

The selective absorption of Ga_2_O_3_ in the UV–C band can advance photonic applications in this important spectral range, such as nonline-of-sight optical communications. To compare the potential of our thin oxides for UV–C sensing, we fabricated devices using both epitaxial GaSe on sapphire and the Ga_2_O_3_ layers obtained by thermal oxidation. Gold contacts were deposited onto the surface of the layers by thermal evaporation through a shadow mask. This consists of a two-terminal interdigitated contact design with a channel width of 50 μm (inset of Figure 5a). Transport studies of devices fabricated from the as-grown (*l* = 75 nm) and annealed layers at *T*_*a*_ = 600 °C (amorphous a-Ga_2_O_3_, see SI5) and *T*_*a*_ = 800 °C (crystalline β-Ga_2_O_3_) in the dark reveal a high resistivity ρ ≈ 1 MΩ·m (insets in Figure 5ai-ii-iii). However, as shown in Figure 5ai-ii-iii, under illumination with UV–C light (λ = 260 nm, *P* = 3.3 μW), the resistivity is reduced to ρ_L_ < 10 kΩ·m, giving a UV–C sensor on/off ratio of ∼10^2^ in devices based on as-grown GaSe and a-Ga_2_O_3_, and >10^3^ for crystalline β-Ga_2_O_3_.

The photoresponsivity *R* of the sensor, calculated as *I*_ph_/*P*, where *I*_ph_ is the photocurrent and *P* is the power incident on the active region of the device, follows a dependence on the excitation wavelength that resembles closely the absorption spectrum of the photoactive layer (Figure S6 in the SI). In particular, the device based on β-Ga_2_O_3_ shows a strong rejection ratio (>10^3^) between UV light at λ = 250 nm and visible light at λ = 400 nm. This aligns well with the transmission spectra in Figure 4a, showing a stronger absorption from Ga_2_O_3_ at photon energies >4.5 eV (λ ∼ 275 nm).

Figure 5b compares the frequency response of the photoresponsivity for the GaSe, a-Ga_2_O_3_ and β-Ga_2_O_3_ detectors under UV–C LED excitation (λ = 265 nm, *P* = 90 μW, *V* = 2 V). The 3 dB bandwidth of the detectors (i.e., frequency range for which the photocurrent amplitude is ≥ 0.707 of its maximum value) is Δ*f* = 170, 60, and 34 Hz for GaSe, a-Ga_2_O_3_ and β-Ga_2_O_3_, respectively. Examples of temporal dependencies of the current under UV–C light are shown in Figure 5c and Figure S6. For all sensors, the temporal modulation of the photocurrent is reproducible over several experiments. From the analysis of the temporal rise and decay of the current, we estimate the rise, τ_r_, and decay, τ_d_, times (Table 1 and Figure S6 in SI). While the GaSe device has the fastest response time and largest Δ*f*, the a-Ga_2_O_3_ and β-Ga_2_O_3_ devices offer the advantage of UV–C selectivity. Additionally, the a-Ga_2_O_3_ and β-Ga_2_O_3_ devices have a larger photoresponsivity under identical experimental conditions than GaSe (Figure 5b-c). Specifically, under the same applied voltage and wavelength of light (λ = 265 nm), a larger photocurrent amplitude is measured in the devices based on β-Ga_2_O_3_. Finally, our prototype thin-film β-Ga_2_O_3_ UV–C sensor can operate at relatively low applied voltages. For example, for *V* = 2 V, *R* is up to 5 mA/W under continuous excitation and *R* = 0.2 mA/W at *f* = 0.5 kHz (λ = 260 nm). We note that the magnitude of the photoresponsivity tends to be weakly dependent on the incident light power, but increases linearly with the applied voltage. The performance parameters of our devices (summarized in Table 1) compare favorably with modern UV–C detectors based on wide band gap semiconductors (see SI5).^23,43^ The large optical absorption of GaSe in the UV–C band and the prospect for its full or partial conversion into a thin layer of Ga_2_O_3_ provide a platform for further advances. In particular, the fast (∼ ms) temporal response of the photocurrent in the GaSe and β-Ga_2_O_3_ devices offers a performance level of interest for optical communication systems in the UV range.

**Table 1:** Comparison of Performance Parameters for GaSe, a-Ga_2_O_3_, and β-Ga_2_O_3_. Detectors under UV–C light (λ = 265 nm, *P* = 90 μW, *f* = 10 Hz) at *V* = 2 V^a^

	τ_r_ (ms)	τ_d_ (ms)	Δ*f* (Hz)	*R* (mA/W)	D* (Jones)
GaSe	1.3	2.9	170	0.1	4.0 × 10^9^
a-Ga_2_O_3_	14.7	12.5	60	0.9	2.4 × 10^9^
β-Ga_2_O_3_	18.6	18.0	34	2.1	1.8 × 10^9^

a Data include the rise and decay times, 3 dB bandwidth Δ*f*, responsivity *R* and specific detectivity D*. Here, D* = (*AΔf*)^1/2^/NEP, where *A* = 8.6 × 10^–7^ m^2^ is the area of the device, NEP = 0.3 nW is the noise equivalent power, and Δ*f* is the 3 dB bandwidth.

## Conclusions

In summary, we have demonstrated the complete conversion of scalable epitaxial thin layers of the van der Waals GaSe crystal into an oxide. Through a dry oxidation process, we have created nanometer-thick crystalline Ga_2_O_3_ layers, with properties that are engineered by the postgrowth thermal oxidation conditions. Optical transmission and Raman spectroscopy provide effective tools to probe the temperature dependence of the thermal oxidation, revealing a clear transition in the optical and vibrational properties of the layers when annealed in oxygen at temperatures greater than 500 °C and up to 900 °C. For the highest annealing temperatures, individual crystalline grains of Ga_2_O_3_ form coherent interfaces with the sapphire substrate, have well-defined crystallographic orientations and undergo a volume contraction, consistent with the higher molecular density of Ga_2_O_3_ compared to that of GaSe. In contrast to previous reports on single GaSe bulk crystals, a full conversion of thin layers of GaSe into an amorphous or crystalline oxide is achieved. The developed method offers opportunities for creating thin-film multifunctional electronic and photonic devices, such as insulating layers and unfiltered UV–C sensors based on amorphous- or crystalline-Ga_2_O_3_. The proposed method is expected to be applicable to other metal chalcogenide two-dimensional semiconductors to facilitate the fabrication of a new range of functional devices via epitaxial and postgrowth thermal oxidation techniques.

## Methods

### MBE Growth

GaSe samples of various thicknesses (24–75 nm) were grown at a rate of ∼1.7 nm/min on 2-inch sapphire substrates using a Scienta Omicron PRO 75 MBE system. Ga and Se were evaporated from effusion cells from Dr. Eberl MBE-Komponenten. The growth was monitored *in situ* by reflection high energy electron diffraction (RHEED). Full details of the substrate preparation and growth conditions are given by Shiffa et al.^12^

### Thermal Annealing

We used a Carbolite Gero TF1 12/125/400 tube furnace. After MBE growth, the samples were diced into small ∼5 × 5 mm^2^ chips for post-growth studies. The size and shape of the diced chips vary due to cracking along preferential directions of the sapphire substrate. The samples were placed in a custom-made quartz boat that sits in the middle of a 3-inch diameter quartz tube. An argon/oxygen gas mixture was used with an argon flow rate of 2 sL/min and oxygen flow rate of 0.5 sL/min using a custom-made baffle to allow sufficient heating of the gas mixture. The temperature ramp rate was controlled in the range 6–30 °C/min and a dwell time at the maximum set temperature between 30 and 240 min was used. The cooling rate was set to 7.5 °C/min. A wide temperature range was examined to identify the required conditions for producing crystalline Ga_2_O_3_. Our preliminary studies and those from the literature show that a structural/chemical change becomes significant at temperatures above 400 °C; also, Ga tends to be desorbed from the substrate at high temperatures (>1000 °C). Different annealing times and ramp rates were examined: the effect of ramp rate was negligible and annealing times >30 min had little effect on the produced material, i.e. the conversion occurs within the first 30 min of annealing in oxygen.

### Optical Transmission and Reflection Spectroscopy

Transmission spectra were acquired by combining an Ocean Optics DH-2000-BAL Deuterium-Halogen LightSource and Ocean Optics UV-NIR Flame-S-XR1-ES miniature spectrometer. The light source was coupled into a fiber and mounted into an alignment stage. A second fiber was used to collect the light into the spectrometer. The samples were placed between the two fibers in order to measure the change in transmission.

### Transmission Electron Microscopy

Using a Titan Cubed3 Themis FEI STEM microscope with double aberration correction, high-resolution high angle annular dark field (HR-HAADF) imaging was conducted at 200 kV in scanning transmission electron microscopy (STEM) mode. This allowed the sample to be examined under HR-STEM conditions with a probe size of 0.2 nm and a spatial resolution ranging from 0.07 to 0.09 nm. The STEM mode used a camera length of 115 mm and a probe convergence semiangle of 21.5 mrad. The HAADF detector gathered electrons scattered within an angle range of 67.6–200 mrad. CrysTBox software was used to identify GaSe and Ga_2_O_3_ polytypes from HRSTEM images (M. Klinger. Institute of Physics of the Czech Academy of Sciences, 2015). The structural models of the interfaces were built using the VESTA software (Ver. 3.5.7).

### Raman Spectroscopy

Raman spectra were acquired under ambient conditions using a Horiba Scientific micro-Raman setup comprising a frequency doubled Nd:YVO4 laser (λ = 532 nm), an *x-y-z* motorized stage, and an optical confocal microscope system (0.5 m-long monochromator and 1200 g mm^–1^ grating). A Si charge-coupled device camera was used for signal detection. The laser beam diameter was focused to ∼1 μm using a 100× objective. Excitation laser powers ranged from 0.23 mW to 23 mW.

### Atomic Force Microscopy

The GaSe layer surface morphology was studied using amplitude modulated tapping mode atomic force microscopy (AFM). An Asylum Research Cypher-S AFM system was used to measure the samples under ambient conditions. The Gwyddion software package was used to process the data.

### Electrical Transport

The electrical properties of the photodetectors were measured with a Keithley 2400 SourceMeter. Illumination in the VIS to UV–C range was provided by a Xe lamp, with the wavelength selected by a HORIBA Jobin Yvon MicroHR monochromator. The power density at each wavelength was measured using a Thorlabs PM100D power meter. All measurements were conducted under vacuum at a pressure of ∼10^–6^ mbar. The frequency response of the photodetectors was measured by illumination with a 265 nm ams OSRAM UVC LED. The LED was electronically modulated with an Aim-TTi TGA1241 50 MHz Arbitrary Waveform Generator amplified by an Aim-TTi WA301 Wideband Amplifier. The voltage output was recorded on a Yokogawa DL850 ScopeCorder, containing a 1 MΩ resistor and a 35 pF capacitor.

### Ellipsometry

A M2000-DI (196 nm–1700 nm) rotating compensator variable angle spectroscopic ellipsometer was used to study the optical properties of the as-grown and oxidized layers. The technique uses focusing probes (minor axis diameter 200 μm) from J.A. Woollam Co. at 55-, 60- and 65-degree angles of incidence. CompleteEase v6.70 was used to model the dielectric functions.

## Data Availability

The data that support the findings of this study are available from the corresponding author upon reasonable request.

## Supporting Information Available

Additional experimental details, materials, and methods (PDF)

## References

[ref1] Novoselov K. S.; Geim A. K.; Morozov S. V.; Jiang D.; Zhang Y.; Dubonos S. V.; Grigorieva I. V.; Firsov A. A. Electric Field Effect in Atomically Thin Carbon Films. Science 2004, 306 (5696), 666–669. 10.1126/science.1102896. 15499015

[ref2] Geim A. K.; Novoselov K. S. The Rise of Graphene. Nat. Mater. 2007, 6 (3), 183–191. 10.1038/nmat1849. 17330084

[ref3] Novoselov K. S.; Mishchenko A.; Carvalho A.; Castro Neto A. H. 2D Materials and van der Waals Heterostructures. Science 2016, 353, 6298 10.1126/science.aac9439. 27471306

[ref4] Huang W.; Gan L.; Li H.; Ma Y.; Zhai T. 2D Layered Group IIIA Metal Chalcogenides: Synthesis, Properties and Applications in Electronics and Optoelectronics. CrystEngComm 2016, 18 (22), 3968–3984. 10.1039/C5CE01986A.

[ref5] Liu Y.; Weiss N. O.; Duan X.; Cheng H. C.; Huang Y.; Duan X. Van der Waals Heterostructures and Devices. Nature Reviews Materials 2016, 1 (9), 1–17. 10.1038/natrevmats.2016.42.

[ref6] Deal B. E.; Grove A. S. General Relationship for the Thermal Oxidation of Silicon. J. Appl. Phys. 1965, 36 (12), 3770–3778. 10.1063/1.1713945.

[ref7] Shi L.; Li Q.; Ouyang Y.; Wang J. Effect of Illumination and Se Vacancies on Fast Oxidation of Ultrathin Gallium Selenide. Nanoscale 2018, 10 (25), 12180–12186. 10.1039/C8NR01533C. 29923588

[ref8] Zheng S.; Li J.; Zhang D.; Zhou Z.; Liu J.; Tao Y.; Fang X.; Yang X.; Han G.; Lu X.; Wang G.; Zhang B.; Wang D.; Zhou X. Assembly of Multisurfaced van der Waals Layered Compound GaSe via Thermal Oxidation. Adv. Funct Mater. 2024, 34, 2309418 10.1002/adfm.202309418.

[ref9] Schmidt C.; Rahaman M.; Zahn D. R. T. Conversion of 2-Dimensional GaSe to 2-Dimensional β-Ga_2_O_3_ by Thermal Oxidation. Nanotechnology 2022, 33 (4), 045702 10.1088/1361-6528/ac2f5d. 34644690

[ref10] Choudhury T. H.; Zhang X.; Al Balushi Z. Y.; Chubarov M.; Redwing J. M. Epitaxial Growth of Two-Dimensional Layered Transition Metal Dichalcogenides. Annu. Rev. Mater. Res. 2020, 50, 155–177. 10.1146/annurev-matsci-090519-113456.

[ref11] Lee C. H.; Krishnamoorthy S.; O’Hara D. J.; Brenner M. R.; Johnson J. M.; Jamison J. S.; Myers R. C.; Kawakami R. K.; Hwang J.; Rajan S. Molecular Beam Epitaxy of 2D-Layered Gallium Selenide on GaN Substrates. J. Appl. Phys. 2017, 121 (9), 94302 10.1063/1.4977697.

[ref12] Shiffa M.; Dewes B. T.; Bradford J.; Cottam N. D.; Cheng T. S.; Mellor C. J.; Makarovskiy O.; Rahman K.; O’Shea J. N.; Beton P. H.; Novikov S. V.; Ben T.; Gonzalez D.; Xie J.; Zhang L.; Patanè A. Wafer-Scale Two-Dimensional Semiconductors for Deep UV Sensing. Small 2024, 20, 2305865 10.1002/smll.202305865. 37798672

[ref13] Liu C. W.; Dai J. J.; Wu S. K.; Diep N. Q.; Huynh S. H.; Mai T. T.; Wen H. C.; Yuan C. T.; Chou W. C.; Shen J. L.; Luc H. H. Substrate-Induced Strain in 2D Layered GaSe Materials Grown by Molecular Beam Epitaxy. Sci. Rep 2020, 10 (12972), 12972 10.1038/s41598-020-69946-4. 32737426 PMC7395717

[ref14] Afaneh T.; Fryer A.; Xin Y.; Hyde R. H.; Kapuruge N.; Gutiérrez H. R. Large-Area Growth and Stability of Monolayer Gallium Monochalcogenides for Optoelectronic Devices. ACS Appl. Nano Mater. 2020, 3 (8), 7879–7887. 10.1021/acsanm.0c01369.

[ref15] Shivani; Kaur D.; Ghosh A.; Kumar M. A Strategic Review on Gallium Oxide Based Power Electronics: Recent Progress and Future Prospects. Mater. Today Commun. 2022, 33, 104244 10.1016/j.mtcomm.2022.104244.

[ref16] Green A. J.; Speck J.; Xing G.; Moens P.; Allerstam F.; Gumaelius K.; Neyer T.; Arias-Purdue A.; Mehrotra V.; Kuramata A.; Sasaki K.; Watanabe S.; Koshi K.; Blevins J.; Bierwagen O.; Krishnamoorthy S.; Leedy K.; Arehart A. R.; Neal A. T.; Mou S.; Ringel S. A.; Kumar A.; Sharma A.; Ghosh K.; Singisetti U.; Li W.; Chabak K.; Liddy K.; Islam A.; Rajan S.; Graham S.; Choi S.; Cheng Z.; Higashiwaki M. β-Gallium Oxide Power Electronics. APL Mater. 2022, 10 (2), 29201 10.1063/5.0060327.

[ref17] Higashiwaki M.; Sasaki K.; Murakami H.; Kumagai Y.; Koukitu A.; Kuramata A.; Masui T.; Yamakoshi S. Recent Progress in Ga_2_O_3_ Power Devices. Semicond. Sci. Technol. 2016, 31 (3), 034001 10.1088/0268-1242/31/3/034001.

[ref18] Kaur D.; Kumar M. A Strategic Review on Gallium Oxide Based Deep-Ultraviolet Photodetectors: Recent Progress and Future Prospects. Adv. Opt Mater. 2021, 9 (9), 2002160 10.1002/adom.202002160.

[ref19] Varshney U.; Aggarwal N.; Gupta G. Current Advances in Solar-Blind Photodetection Technology: Using Ga_2_O_3_ and AlGaN. J. Mater. Chem. C Mater. 2022, 10 (5), 1573–1593. 10.1039/D1TC05101F.

[ref20] Pearton S. J.; Yang J.; Cary P. H.; Ren F.; Kim J.; Tadjer M. J.; Mastro M. A. A Review of Ga_2_O_3_ Materials, Processing, and Devices. Appl. Phys. Rev. 2018, 5 (1), 11301 10.1063/1.5006941.

[ref21] Yuan Y.; Hao W.; Mu W.; Wang Z.; Chen X.; Liu Q.; Xu G.; Wang C.; Zhou H.; Zou Y.; Zhao X.; Jia Z.; Ye J.; Zhang J.; Long S.; Tao X.; Zhang R.; Hao Y. Toward Emerging Gallium Oxide Semiconductors: A Roadmap. Fundamental Research 2021, 1 (6), 697–716. 10.1016/j.fmre.2021.11.002.

[ref22] Vavoulas A.; Sandalidis H. G.; Chatzidiamantis N. D.; Xu Z.; Karagiannidis G. K. A Survey on Ultraviolet C-Band (UV-C) Communications. IEEE Communications Surveys and Tutorials 2019, 21 (3), 2111–2133. 10.1109/COMST.2019.2898946.

[ref23] Xie C.; Lu X.-T.; Tong X.-W.; Zhang Z.-X.; Liang F.-X.; Liang L.; Luo L.-B.; Wu Y.-C. Recent Progress in Solar-Blind Deep-Ultraviolet Photodetectors Based on Inorganic Ultrawide Bandgap Semiconductors. Adv. Funct Mater. 2019, 29 (9), 1806006 10.1002/adfm.201806006.

[ref24] Yoshioka S.; Hayashi H.; Kuwabara A.; Oba F.; Matsunaga K.; Tanaka I. Structures and Energetics of Ga_2_O_3_ Polymorphs. J. Phys.: Condens. Matter 2007, 19 (34), 346211 10.1088/0953-8984/19/34/346211.

[ref25] Tak B R; Kumar S.; Kapoor A K; Wang D.; Li X.; Sun H.; Singh R Recent Advances in the Growth of Gallium Oxide Thin Films Employing Various Growth Techniques—a Review. J. Phys. D Appl. Phys. 2021, 54 (45), 453002 10.1088/1361-6463/ac1af2.

[ref26] Abejide F. H.; Ajayi A. A.; Akinsola S. I.; Alabi A. B. Properties of Gallium Oxide Thin Film Prepared on Silicon Substrate by Spray Pyrolysis Method. J. Mater. Sci. 2022, 57 (45), 21135–21142. 10.1007/s10853-022-07952-9.

[ref27] Tomm Y.; Reiche P.; Klimm D.; Fukuda T. Czochralski Grown Ga_2_O_3_ Crystals. J. Cryst. Growth 2000, 220 (4), 510–514. 10.1016/S0022-0248(00)00851-4.

[ref28] Galazka Z. Growth of Bulk β-Ga_2_O_3_ Single Crystals by the Czochralski Method. J. Appl. Phys. 2022, 131 (3), 31103 10.1063/5.0076962.

[ref29] Kuramata A.; Koshi K.; Watanabe S.; Yamaoka Y.; Masui T.; Yamakoshi S. High-Quality β-Ga_2_O_3_ Single Crystals Grown by Edge-Defined Film-Fed Growth. Jpn. J. Appl. Phys. 2016, 55 (12), 1202A2 10.7567/JJAP.55.1202A2.

[ref30] Hoshikawa K.; Ohba E.; Kobayashi T.; Yanagisawa J.; Miyagawa C.; Nakamura Y. Growth of β-Ga_2_O_3_ Single Crystals Using Vertical Bridgman Method in Ambient Air. J. Cryst. Growth 2016, 447, 36–41. 10.1016/j.jcrysgro.2016.04.022.

[ref31] Ghose S.; Rahman Md. S.; Rojas-Ramirez J. S.; Caro M.; Droopad R.; Arias A.; Nedev N. Structural and Optical Properties of β-Ga_2_O_3_ Thin Films Grown by Plasma-Assisted Molecular Beam Epitaxy. Journal of Vacuum Science & Technology B: Nanotechnology and Microelectronics 2016, 34 (2), 02L109 10.1116/1.4942045.

[ref32] Lv Y.; Ma J.; Mi W.; Luan C.; Zhu Z.; Xiao H. Characterization of β-Ga_2_O_3_ Thin Films on Sapphire (0001) Using Metal-Organic Chemical Vapor Deposition Technique. Vacuum 2012, 86 (12), 1850–1854. 10.1016/j.vacuum.2012.04.019.

[ref33] Zhuo Y.; Chen Z.; Tu W.; Ma X.; Pei Y.; Wang G. β-Ga_2_O_3_ versus ε-Ga_2_O_3_: Control of the Crystal Phase Composition of Gallium Oxide Thin Film Prepared by Metal-Organic Chemical Vapor Deposition. Appl. Surf. Sci. 2017, 420, 802–807. 10.1016/j.apsusc.2017.05.241.

[ref34] Zhang B. Y.; Xu K.; Yao Q.; Jannat A.; Ren G.; Field M. R.; Wen X.; Zhou C.; Zavabeti A.; Ou J. Z. Hexagonal Metal Oxide Monolayers Derived from the Metal-Gas Interface. Nat. Mater. 2021, 20 (8), 1073–1078. 10.1038/s41563-020-00899-9. 33462466

[ref35] Zhang Y.; He Q.; Yang H.; Li Z.; Jiang H.; Zhang Y.; Luo X.; Zheng Y. Liquid-Metal-Based Spin-Coating Exfoliation for Atomically Thin Metal Oxide Synthesis. Nano Lett. 2024, 24 (21), 6247–6254. 10.1021/acs.nanolett.4c00757. 38709758

[ref36] Quan S.; Wang Y.; Jiang J.; Fu S.; Li Z.; Liang Y.; Guo S.; Zhong B.; Yu K.; Zhang H.; Kan G. Photo-Oxidation Dynamics in GaSe Flakes Probed through Temporal Evolution of Raman Spectroscopy. J. Phys. Chem. C 2021, 125 (46), 25608–25614. 10.1021/acs.jpcc.1c03708.

[ref37] Guerrero E.; Strubbe D. A. Computational Generation of Voids in *a*-Si and *a*-Si:H by Cavitation at Low Density. Phys. Rev. Mater. 2020, 4 (2), 025601 10.1103/PhysRevMaterials.4.025601.

[ref38] Ho C.-H.; Lai X.-R.; Chuang C.-A.; Kuo W.-L.; Tiong K.-K. The Study of Optical Properties of III_2_-VI_3_ Defect Semiconductor Group Compounds Ga_2_S_3_, Ga_2_Se_3_, In_2_S_3_, and In_2_Se_3_. Adv. Photonics Res. 2021, 2 (3), 2000110 10.1002/adpr.202000110.

[ref39] Goldan A. H.; Li C.; Pennycook S. J.; Schneider J.; Blom A.; Zhao W. Molecular Structure of Vapor-Deposited Amorphous Selenium. J. Appl. Phys. 2016, 120 (13), 135101 10.1063/1.4962315.

[ref40] Beechem T. E.; Kowalski B. M.; Brumbach M. T.; McDonald A. E.; Spataru C. D.; Howell S. W.; Ohta T.; Pask J. A.; Kalugin N. G. Oxidation of Ultrathin GaSe. Appl. Phys. Lett. 2015, 107 (17), 173103 10.1063/1.4934592.

[ref41] Siciliano T.; Tepore M.; Genga A.; Micocci G.; Siciliano M.; Tepore A. Thermal Oxidation of Amorphous GaSe Thin Films. Vacuum 2013, 92, 65–69. 10.1016/j.vacuum.2012.12.001.

[ref42] Gerlach G.; Maser K. A Self-Consistent Model for Thermal Oxidation of Silicon at Low Oxide Thickness. Advances in Condensed Matter Physics 2016, 2016, 7545632 10.1155/2016/7545632.

[ref43] Shu L.; Yao S.; Xi Z.; Liu Z.; Guo Y.; Tang W. Multi-Pixels Gallium Oxide UV Detector Array and Optoelectronic Applications. Nanotechnology 2024, 35 (5), 052001 10.1088/1361-6528/ad079f. 37890476

