# Supplementary material for: Thin Ga2O3 Layers by Thermal Oxidation of van der Waals GaSe Nanostructures for Ultraviolet Photon Sensing
Source: ACS Appl Nano Mater. 2024 Jul 31;7(15):17553–60. doi: 10.1021/acsanm.4c02685 (PMC11320379; doi:10.1021/acsanm.4c02685)
Supplement: Supplementary file 1 — an4c02685_si_001.pdf [file an4c02685_si_001.pdf]

Supporting Information

Thin Ga_2O_3 Layers by Thermal Oxidation of van der Waals GaSe Nanostructures for Ultraviolet Photon Sensing

Nathan D. Cottam[1*] Benjamin T. Dewes,[1] Mustaqeem Shiffa,[1] Tin S. Cheng,[1] Sergei V. Novikov,[1] Christopher J. Mellor,[1] Oleg Makarovsky,[1] David Gonzalez,[2] Teresa Ben,[2] Amalia Patanè[1*]

[1]School of Physics and Astronomy, University of Nottingham, Nottingham NG7 2RD, UK
Email Address: amalia.patane@nottingham.ac.uk
nathan.cottam@nottingham.ac.uk

[2]University Research Institute on Electron Microscopy and Materials, IMEYMAT, Universidad de Cadiz, 11510 Cadiz, Spain

Table of contents

SI1. TEM and EDX analyses

SI2. Modelling the conversion of GaSe into an oxide

SI3. Atomic force microscopy

SI4. Ellipsometry

SI5. Photon sensor based on conversion of GaSe into Ga_2O_3

SI1. TEM and EDX analyses

Cross-sectional scanning transmission electron microscopy (STEM) images of an as-grown 75 nm-thick GaSe layer (**Figure S1a**) and the produced oxide after annealing at 900 °C in an oxygen-argon atmosphere (**Figure S1b**). They show an average thickness reduction of ~15%, from 75 nm to 62-69 nm due to the conversion of GaSe into β-Ga_2O_3. The oxide shows high crystallinity and uniformity near the substrate interface and at the surfaces of the layers. However, nanoscale voids are present throughout the central regions. These are assigned to the contraction of the crystal lattice as the material is converted from GaSe to β-Ga_2O_3.

Figures S2 and **S3** present representative high-angle annular dark-field (HAADF) images and energy-dispersive X-Ray (EDX) spectroscopy data of the GaSe and oxide interfaces, respectively. In **Figure S2**, the alternating Ga and Se layers observed suggest a well-ordered epitaxial growth of the GaSe layers. In **Figure S3**, the absence of a Se signal above the sapphire substrate in elemental quantification is consistent with the detection of only Ga and O in a 2/3 ratio, indicative of Ga_2O_3 formation in the sample annealed at T_a = 600 °C.

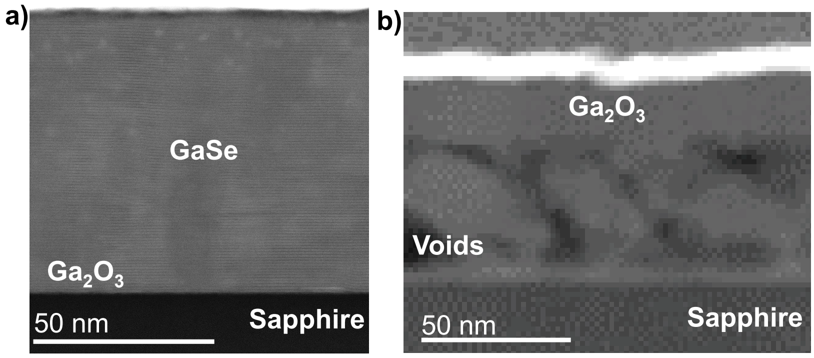

Figure S1. Cross-sectional STEM images of a) as grown GaSe (nominal thickness of 75 nm) and b) of the same material annealed at 900 °C in an oxygen-argon atmosphere.

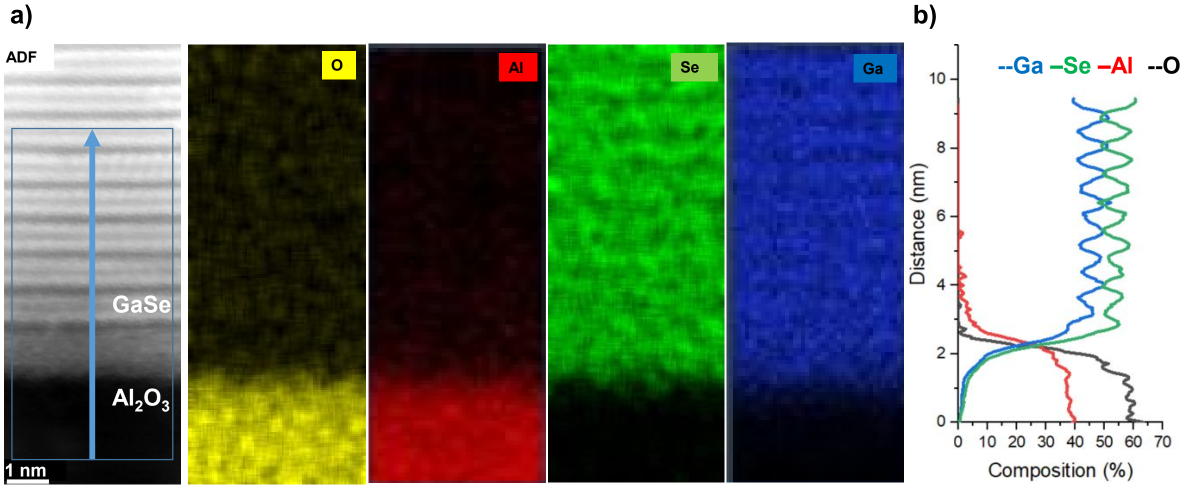

Figure S2. a) HAADF image of the GaSe/sapphire interface and the EDX elemental maps of O, Al, Se and Ga by analyzing their characteristic X-ray peaks. b) Average line composition profile along the growth direction from the region inside the blue rectangle.

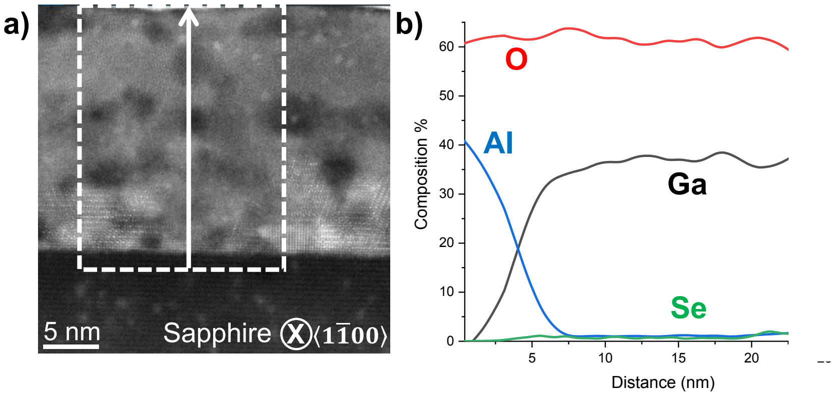

Figure S3. a) ADF-STEM image of a sample annealed at 600 °C in an oxygen-argon atmosphere. b) Average EDX profile extracted from the region inside the white rectangle of the EDX map in part a). The [Ga]/[O] ratio corresponds to a stoichiometric ratio of 2/3. The measured Se signal is negligible within the experimental error of the technique.

SI2. Modelling the conversion of GaSe into an oxide

GaSe and Ga_2O_3 have different molecular weights and crystal densities. Thus, when converting GaSe into Ga_2O_3, we expect a change in the volume of the material. If we assume that no gallium atoms are lost in the conversion, the molar ratio of GaSe:Ga_2O_3 must be 2:1 in order to conserve the number of Ga atoms. For simplicity, let's consider 2 moles of GaSe, which will return 1 mole of Ga_2O_3. GaSe has a molar mass of 148.69 g/mol and a density of 5.03 g/cm^3, therefore 2 moles of GaSe occupies a volume of 59.1 cm^3. Likewise, 1 mole of Ga_2O_3 has a molar mass of 187.44 g/mol and a density of 5.88 g/cm^3, giving a volume of 31.9 cm^3. This gives a contraction in the volume of ~46% i.e. $l_{Ga2O3} \sim 0.54l$, where l is the nominal thickness of GaSe. Here, molar mass and density values for GaSe and β-Ga_2O_3 were obtained from the PubChem[1] and American Elements[2] databases.

SI3. Atomic force microscopy

The MBE-grown GaSe layers were imaged by atomic force microscopy (AFM) in ambient conditions before and after annealing at various temperatures (**Figure S4**). The pristine sample surface consists of pyramid-shaped islands with typical widths of ~ 100 nm, indicative of a dominant spiral growth mode, as previously reported (**Figure S4a**).[3] After annealing at 500 °C, the islands become less well ordered, losing the distinct atomic steps in the pristine sample as the layers are converted to Ga_2Se_3 (**Figure S4b**). The surface of the amorphous gallium oxide produced by annealing at 600 °C comprises clusters with diameters of 50 - 100 nm (**Figure S4c**). Increasing the temperature to 800 °C to form crystalline Ga_2O_3 instead creates ribbon-like clusters with diameters of ~ 20 - 50 nm (**Figure S4d**). In general, no clear surface order is preserved from the conversion of the GaSe layers into Ga_2O_3.

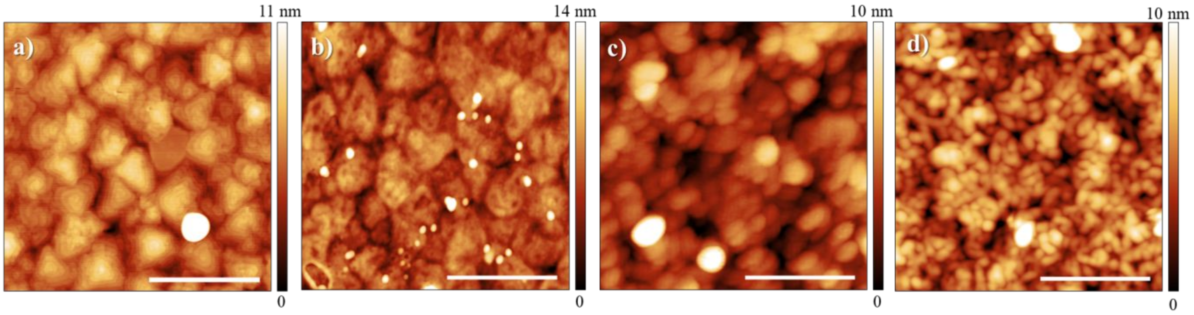

Figure S4. Ambient AFM images of GaSe layers (nominal thickness 75 nm) a) before annealing and after annealing at b) 500 °C, c) 600 °C and d) 800 °C (scale bars = 400 nm).

SI4. Ellipsometry

We have used variable angle spectroscopic ellipsometry (VASE) to obtain the optical constants (refractive index in **Figure S5a** and extinction coefficient in **Figure S5b**) of the as-grown GaSe layers (l = 75 nm) and the oxides formed at T_a = 700 °C and 800 °C. Ellipsometry measures the change in state of polarization of polarized light upon reflection from a surface. The phase difference (Δ) and amplitude ratio (Ψ) are related to the optical reflectance ratio, r, between the p- and s-polarization of light, $r_p/r_s = \tan(\Psi)e^{i\Delta}$. The optical model that fits the data well consists of an upper layer, that is homogeneous, and a lower layer that is mostly void, with a fraction of voids of about 80%. The total thickness of the layers is around 56 nm (T_a = 800 °C) and 63 nm (T_a = 700 °C) which is thinner than the layer thickness (75 nm) of the as-grown GaSe layer. The two annealed samples were measured at three positions on each sample, revealing similar properties.

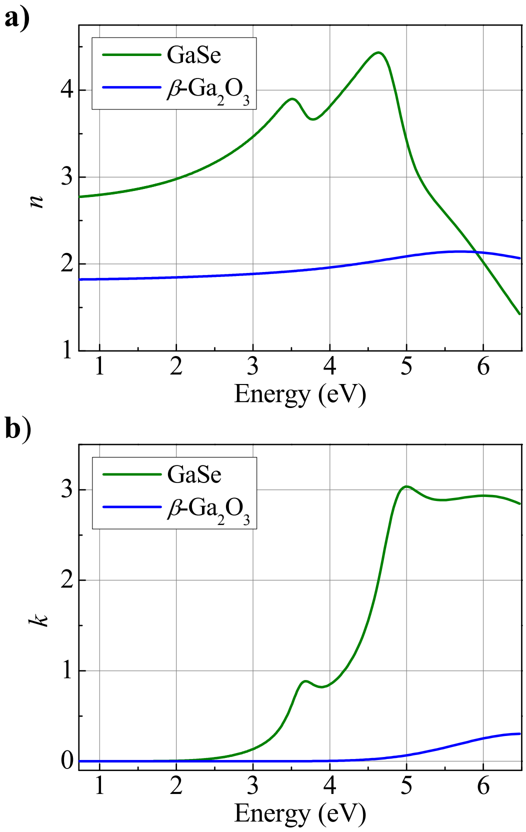

Figure S5 Optical constants. a) Refractive index, n and b) extinction coefficient, k, as determined by spectroscopic ellipsometry for as-grown GaSe (l = 75 nm) and for the same material annealed in oxygen at T_a = 800 °C to form β-Ga_2O_3.

SI5. Photon sensor based on conversion of GaSe into Ga_2O_3

Figure S6a compares the normalised photoresponsivity versus excitation wavelength for GaSe and amorphous and crystalline Ga_2O_3. The sensors based on Ga_2O_3 demonstrate selective response in the UV-C range whilst the GaSe sensor is sensitive over the wide UV and visible spectral range. **Figure S6b-c-d** shows typical temporal response curves for the photocurrent under UV-C light and the estimation of the rise, τ_r, and decay, τ_d, times of the detectors using the time taken between 10% and 90% of the maximum photocurrent signal. Similar values of τ_r and τ_d were obtained by exponential fits to the photocurrent.

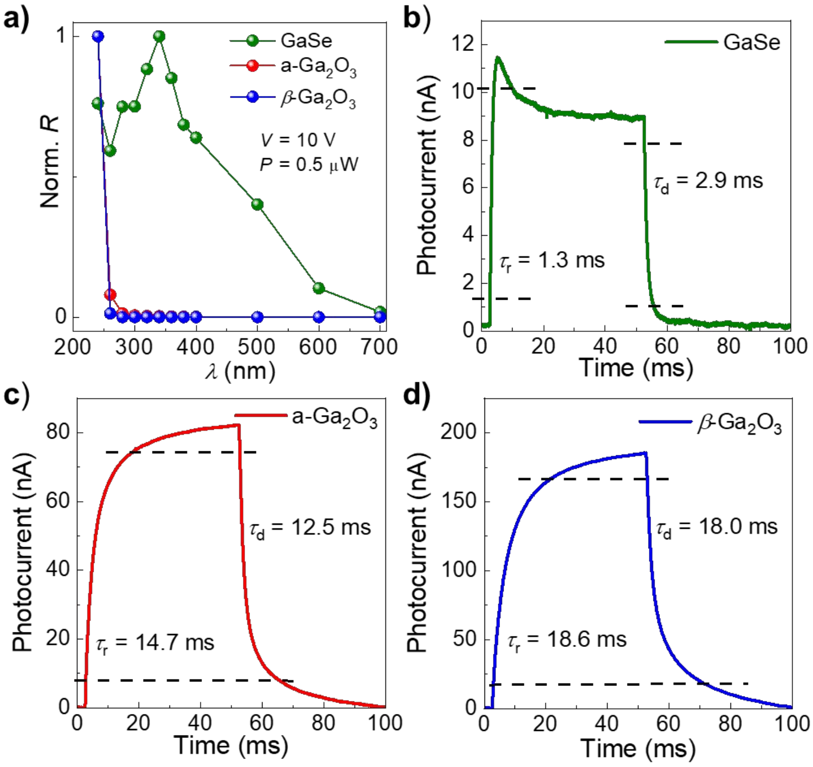

Figure S6. a) A comparison of the normalised responsivity R versus λ for GaSe (green), a-Ga_2O_3 (red) and β-Ga_2O_3 (blue). (b-c-d) Temporal response of the devices in part (a) under UV-C LED excitation ($\lambda = 265$ nm, $P = 90$ μW, $V = 2$ V) for b) GaSe, c) a-Ga_2O_3 and d) β-Ga_2O_3. Dashed black lines denote 10% and 90% of the photocurrent, which is used for calculating response times.

Table S1 shows a summary of the photodetector performance parameters of the β-Ga_2O_3 device developed in this work compared with other similar devices in the recent literature. We note that most reports on "thin films" of Ga_2O_3 in the literature are considerably thicker than our converted layers. Also, the photoresponse is normally reported under larger applied voltages. An important property of our devices is their relatively fast temporal response (~ 19 ms) in the UV-C band, crucial for the operation of a communications system in the UV-C band.

Ref.	Material	Thickness (nm)	Band Gap (eV)	On/Off ratio	R (mA/W)	D^* (Jones)	τ_r/τ_d (s)
**This work**	β-Ga_2O_3 ($V = 2$ V)	< 75	4.5	10^5	5	1.8×10^9	$(19/18)\times10^{-3}$
**[4]**	Ga_2O_3 ($V = 10$ V)	-	4.9	4.3×10^4	89	4.8×10^{10}	0.5/0.7
**[5]**	SiO_2/β-Ga_2O_3 heterojunction ($V = 10$ V)	213	4.9	4×10^2	3.3×10^5	10^{13} ^	$(19.5/9.1)\times10^{-2}$
**[6]**	a-Ga_2O_3 ($V = 10$ V)	100	4.8	> 100	7×10^4	1.3×10^{14} ^	2.3/0.1
**[7]**	α-Ga_2O_3 ($V = 20$ V)	~ 700	5.3	2.8×10^4	5×10^5	4.7×10^{15}	5.47/0.44
**[8]**	β-Ga_2O_3/GaN ($V = -5$ V)	20-500	-	2.3×10^4	2×10^3	1.7×10^{11}	0.22/0.11

Table S1. Comparison of device performance in this work for thin Ga_2O_3 and of similar devices in literature based on thicker layers. The comparison is limited to the UV-C spectral range. In the Table the symbol ^ denotes D^* calculated from dark current assuming that the shot noise is the dominant noise rather than 3dB bandwidth, as measured in this work.

References

1. PubChem: https://pubchem.ncbi.nlm.nih.gov [Accessed 1st May 2024].
2. American Elements: https://www.americanelements.com [Accessed 1st May 2024].
3. Shiffa, M.; Dewes, B. T.; Bradford, J.; Cottam, N. D.; Cheng, T. S.; Mellor, C. J.; Makarovskiy, O.; Rahman, K.; O'Shea, J. N.; Beton, P. H.; Novikov, S. V.; Ben, T.; Gonzalez, D.; Xie, J.; Zhang, L.; Patanè, A. Wafer-Scale Two-Dimensional Semiconductors for Deep UV Sensing. *Small* **2023**, *20*, 2305865.
4. Tsay, C.-Y.; Tsai, H.-M.; Sittimart, P.; Sreenath, M. V.; Kusaba, T.; Yoshitake, T. *30th International Workshop on Active-Matrix Flatpanel Displays and Devices (AM-FPD), Kyoto, Japan* **2023**, 230-231.
5. Liu, Z.; Li, S.; Yan, Z.; Liu, Y.; Zhi, Y.; Wang, X.; Wu, Z.; Li, P.; Tang, W. *J. Mater. Chem. C* **2020**, 8, 5071-5081.
6. Qian, L.-X.; Wu, Z.-H.; Zhang, Y.-Y.; Lai, P. T.; Liu, X.-Z.; Li, Y.-R. *ACS Photonics* **2017**, 4, 2203–2211.
7. Kim, S.; Yoon, Y.; Seo, D.; Park, J.-H.; Jeon, D.-W.; Hwang, W. S.; Shin, M. *APL Mater.* **2023**, 11, 061107.8. Wang, J.; Ji, X.; Yan, Z.; Yan, X.; Lu, C.; Li, Z.; Qi, S.; Li, S.; Qi, X.; Zhang, S.; Hu, S.; Li, P. *Mater. Sci. Semicond. Process.* **2023**, 159, 107372.
